# Deep learning approach for discrimination of liver lesions using nine time-phase images of contrast-enhanced ultrasound

**DOI:** 10.1007/s10396-023-01390-z

**Published:** 2023-12-05

**Authors:** Naohisa Kamiyama, Katsutoshi Sugimoto, Ryuichi Nakahara, Tatsuya Kakegawa, Takao Itoi

**Affiliations:** 1 Ultrasound General Imaging, GE HealthCare Japan, 127 Asahigaoka-4, Hino, Tokyo 191-0065 Japan; 2 Department of Gastroenterology and Hepatology, Tokyo Medical University, Tokyo, 160-0023 Japan; 3 Department of Orthopedic Surgery, Dentistry and Pharmaceutical Sciences, Okayama University Graduate School of Medicine, Okayama, 700-8558 Japan

**Keywords:** Hepatocellular carcinoma, Contrast-enhanced ultrasonography, Machine learning, Multi-input deep learning model

## Abstract

**Purpose:**

Contrast-enhanced ultrasound (CEUS) shows different enhancement patterns depending on the time after administration of the contrast agent. The aim of this study was to evaluate the diagnostic performance of liver nodule characterization using our proposed deep learning model with input of nine CEUS images.

**Methods:**

A total of 181 liver lesions (48 benign, 78 hepatocellular carcinoma (HCC), and 55 non-HCC malignant) were included in this prospective study. CEUS were performed using the contrast agent Sonazoid, and in addition to B-mode images before injection, image clips were stored every minute up to 10 min. A deep learning model was developed by arranging three ResNet50 transfer learning models in parallel. This proposed model allowed inputting up to nine datasets of different phases of CEUS and performing image augmentation of nine images synchronously. Using the results, the correct prediction rate, sensitivity, and specificity between “benign” and “malignant” cases were analyzed for each combination of the time phase. These accuracy values were also compared with the washout score judged by a human.

**Results:**

The proposed model showed performance superior to the referential standard model when the dataset from B-mode to the 10-min images were used (sensitivity: 93.2%, specificity: 65.3%, average correct answer rate: 60.1%). It also maintained 90.2% sensitivity and 61.2% specificity even when the dataset was limited to 2 min after injection, and this accuracy was equivalent to or better than human scoring by experts.

**Conclusion:**

Our proposed model has the potential to identify tumor types earlier than the Kupffer phase, but at the same time, machine learning confirmed that Kupffer-phase Sonazoid images contain essential information for the classification of liver nodules.

## Introduction

Contrast-enhanced ultrasound (CEUS) is a valuable imaging modality for characterizing hepatic nodules without renal toxicity and radiation exposure. Also, due to its real-time imaging nature, CEUS can overcome the issues in the arterial phase of CT or MRI, with a higher sensitivity for detecting arterial hyperenhancement [1, 2]. Nowadays, there are two types of US contrast agents available for clinical use: pure intravascular agents and Kupffer cell agents. Pure blood agents include SonoVue or Lumason (Bracco Diagnostics, Monroe Township, NJ, USA) and Definity (Lantheus Medical Imaging, Billerica, MA, USA), and combined blood pool and Kupffer cell contrast agents include Sonazoid (GE HealthCare, Amersham, UK). As for SonoVue, which is the most-used US contrast agent in the world, CEUS using SonoVue is recommended as a second-line diagnostic modality (i.e., after CT or MRI) for diagnosing hepatocellular carcinoma (HCC) in the European Association for the Study of the Liver (EASL) guidelines [3]. And CEUS with pure intravascular agents can be used to differentiate HCC from other hepatic nodules by using strict criteria of arterial phase hyperenhancement (APHE) and mild washout with later onset (≥ 60 s) [4]. In comparison, Sonazoid has only been available in a few countries such as Japan, South Korea, China, and Norway. Sonazoid bubbles are mainly taken up by Kupffer cells in the liver and demonstrate so-called “Kupffer phase” [5]. Thus, Sonazoid provides not only vascular imaging like a SonoVue but also yields sustained liver parenchymal enhancement for at least 1 h [6]. According to the Asian Pacific Association for the Study of the Liver, the Japan Society of Hepatology, and the Korean Liver Cancer Association [2, 7, 8], CEUS using Sonazoid is also recommended as a second-line diagnostic modality for diagnosing HCC. They proposed APHE and Kupffer phase hypoenhancement as the diagnostic criteria for HCC [9].

Some reports compared the diagnostic performance of CEUS with SonoVue and with Sonazoid for liver nodules, which mainly concluded that CEUS with Sonazoid was non-inferior to that with SonoVue [10, 11]. These reports discussed improving the accuracy of diagnosing the target lesion as malignant or benign, which is another important classification of liver tumors. However, from the viewpoint of time efficiency, CEUS with Sonazoid may be less efficient than that with SonoVue because CEUS with Sonazoid takes more time as we have to wait until the Kupffer phase, which is defined as 10 min after contrast injection. Thus, a method to differentiate such liver nodules in an earlier phase of the CEUS examination is beneficial.

Machine learning approaches have been widely investigated in the medical field. In particular, deep learning is one of the most efficient schemes, and several studies using ultrasound liver images have been reported [12–20]. These prior reports suggest that deep learning has great potential to recognize patterns in medical images, especially complicated patterns that are difficult to differentiate or generalize with the human eye. CEUS may be a good candidate for the deep learning approach because the enhanced image patterns drastically vary due to time after injection of the contrast agent, and these patterns are different among the different types of nodules [14–16]. Also, if a deep learning method can differentiate the CEUS image pattern in an earlier phase, it will contribute to reducing the duration of the ultrasound examination. The aim of the present study was to propose a deep learning model that enables us to input different time phases of CEUS images to evaluate the diagnostic performance for differentiating liver nodules, and to consider the diagnostic information in each time phase.

## Materials and methods

This study was reviewed and approved by Tokyo Medical University’s ethics review board, and written informed consent was obtained from all participants. The diagnostic US scanner used (LOGIQ E10; GE HealthCare, Wauwatosa, WI, USA) was provided by the manufacturer. Only authors with no conflicts of interest had full control of the inclusion of data or information.

### Participants

Between June 2020 and January 2023, 219 patients who had a treatment-naïve hepatic nodule (≥ 1 cm) were consecutively recruited at Tokyo Medical University Hospital. The inclusion criteria were as follows: (a) age 20 years or older, (b) at least one treatment-naïve hepatic nodule (≥ 1 cm), (c) all nodules were visible at baseline US. When multiple eligible lesions were detected, one representative lesion per patient was analyzed.

### US examination

**Fig. 1:**
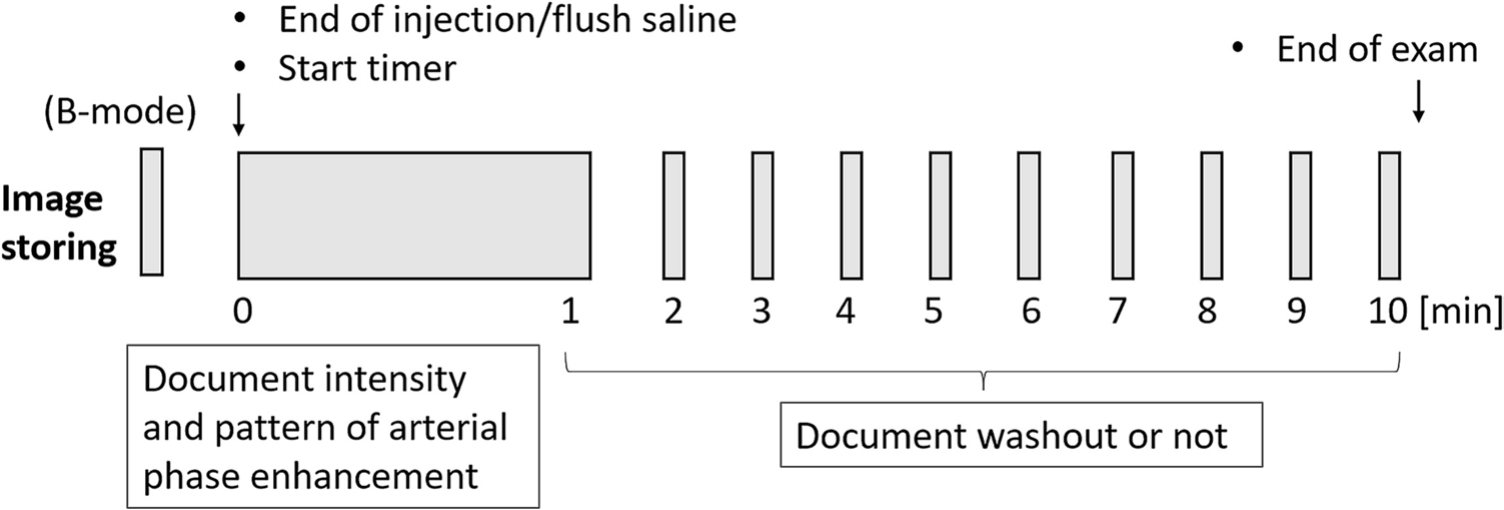
Sonazoid contrast-enhanced ultrasound protocol

Conventional grayscale and CEUS examinations were performed by one of two hepatologists (K.S and H.T with 15 and 5 years of experience with abdominal US, respectively) using an ultrasound scanner (LOGIQ E10 Series) with a convex transducer (C1-6-D, 3.5 MHz center frequency). The imaging mode for CEUS was the amplitude modulation method with a low mechanical index (MI) of 0.16–0.2 and a dynamic range of 63 dB. The Sonazoid contrast agent was injected as a 0.5 mL bolus into an antecubital vein via a 21-gauge peripheral intravenous cannula, followed by a 10 mL saline flush. A timer was started at the time of contrast agent injection. The targeted lesion was recorded continuously as a cine clip for 60 s after injection. During this period, the patient was instructed to breathe gently. After that, the same targeted lesion was recorded at 1 min intervals as a 5 s cine clip with breath-hold. This occurred between the 2 min mark and the 10 min mark. The sequence that was followed in the CEUS protocol is shown in Fig. 1.

### Image dataset

The input images are based on the dataset shown in the “US examination” section. All cine clips were transferred to a PC, and areas of the lesion were extracted with dedicated software programmed in Python. The extraction steps are as follows: Open the image, draw a square region of interest (ROI), and save the extracted image. The size of the ROI can be varied from case to case so that it is approximately 50% larger than the diameter of the target lesion. The ROI aspect ratio was fixed to 1:1 (square), and the size was fixed within the same case.

**Fig. 2:**
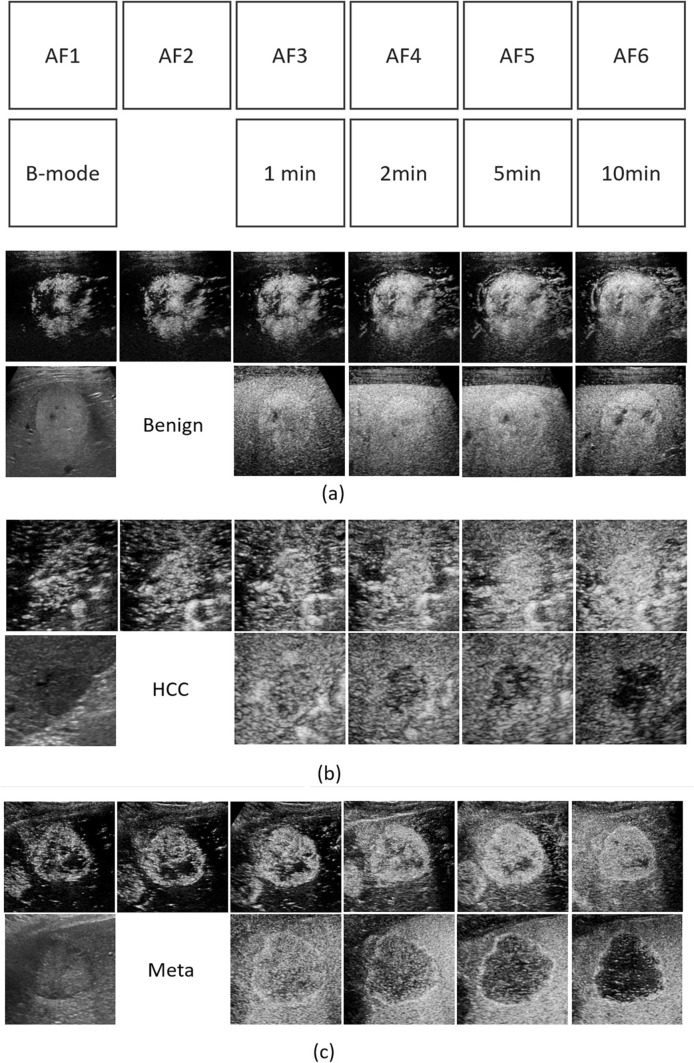
Examples of the extracted images. **a** Benign, **b** HCC, and **c** non-HCC malignant tumor. The time phase of each image corresponds to the caption on the upper right

The time phases of the extracted image are B-mode before contrast injection, arterial-flow phase (AF), and 1, 2, 5, and 10 min after injection. One still image was used for each phase, except for AF, where six images were extracted from the cine clip for 60 s after injection. These six images were manually selected by the investigators to contain the dynamic variation of the enhanced patterns. During this period, the patient breathes gently, so the position of the nodule changes slightly. Therefore, we selected images with a reliable maximum diameter that appeared in the respiratory cycle. The duration was around from 20 to 40 s after injection. Examples of extracted images for each phase are shown in Fig. 2. This study grouped these data into three categories: (1) benign (including hemangiomas, FNH, and AML), (2) HCC, and (3) non-HCC malignant (including metastasis, ICC, CCC).

### Observer study

The ROC observer study was conducted by five hepatologists (two with more than 15 years, and three with less than 10 years of experience) in liver CEUS using free ROC software (ROCViewer-ForMethod1ver.1.0.1) developed by the Japanese Society of Radiological Technology (JSRT) task group. The software provided a rating bar for determining the confidence of differentiation of benign or malignant by clicking the mouse on the bar, where the left and right ends corresponded to definitely benign and definitely malignant, respectively. Rating scales were then converted with continuous values from 0.0 to 1.0 using the distance from the left end of the rating bar to the point clicked. We provided four independent reading sessions including the following image sets: 1, B-mode images; 2, B-mode + AF + 2-min images; 3, B-mode + AF + 2-min + 5-min images; 4, B-mode + AF + 2-min + 5-min + 10-min images. Before the observer study, each reader was instructed how to use the software’s rating bar. The criteria they used to determine the confidence of differentiation of benign or malignant were based on the ultrasound diagnostic criteria for hepatic tumors [21]. Cutoff values were determined by ROC analysis using the Youden index.

Furthermore, one hepatologist who did not participate in the observer study classified all nodules into three patterns such as hyper, iso, or hypo based on their degree of intensity relative to liver parenchymal intensity at each phase (i.e., AF, 2 min, 5 min, and 10 min).

### Deep learning model

**Fig. 3:**
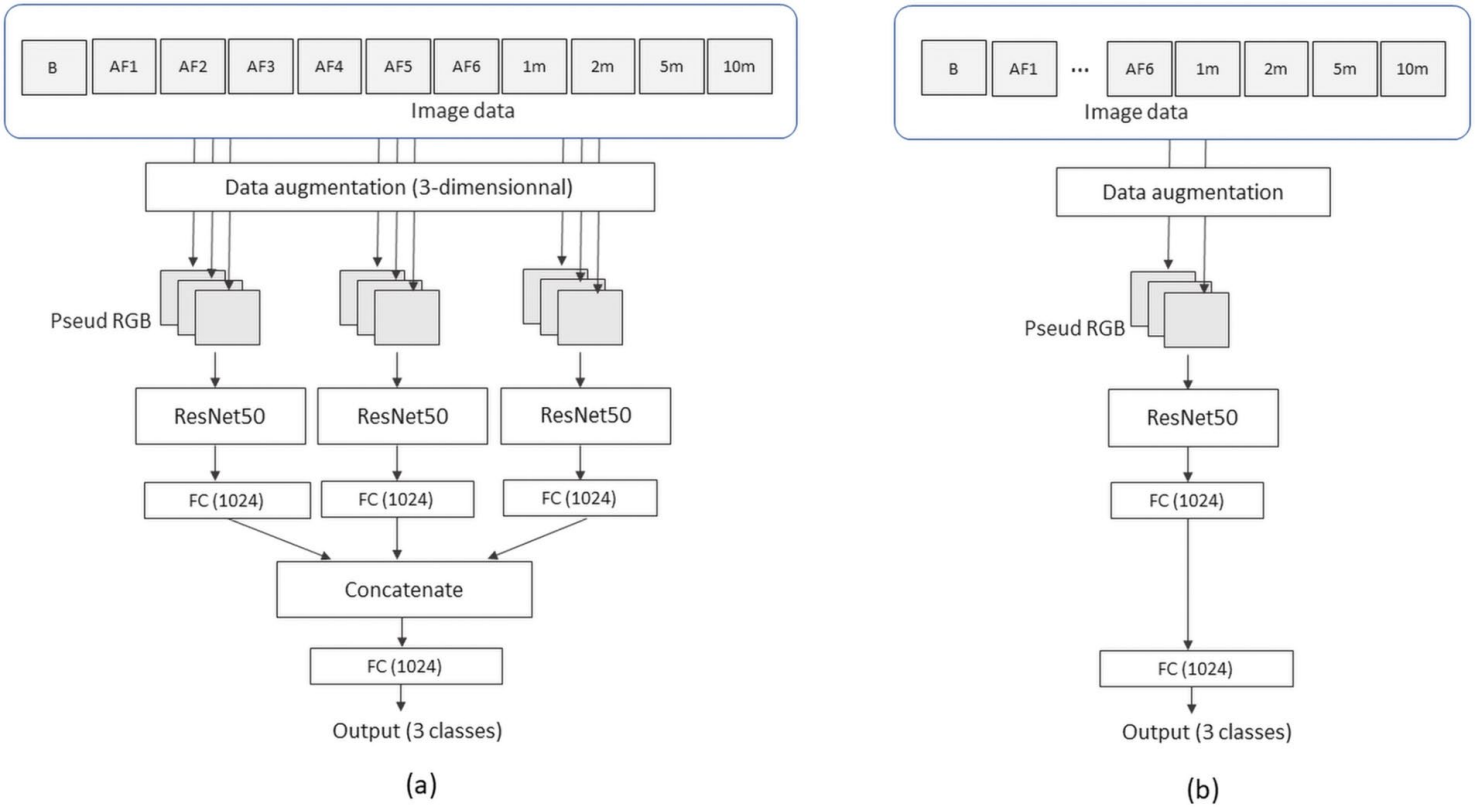
Conceptual flow chart of the CNN used in this study. The transfer learning model ResNet50 accepts three grayscale images as input. Three ResNet50s are concatenated in a way that synchronizes the data extension. (*FC* full connection). **a** Our proposed nine-input model and **b** one pseudo-RGB image input model for comparison

Deep learning was performed using a PC with a GeForce RTX 3060 12 GB GDDR6 GPU (NVIDIA, Santa Clara, CA) and a Core i5-12400 4.40-GHz CPU (Intel, Santa Clara, CA). The software was written with Python 3.8.5 and TensorFlow 2.4.1. Image data were separated at a ratio of 9:1 for training and testing, respectively. The training image set was split at a ratio of 8:2 for training and validation. These were randomized but split evenly among the three categories. We used ResNet50 for transfer learning. Figure 3A shows a conceptual flow chart of the deep learning model in this study, which is based on a convolutional neural network (CNN) model. ResNet50 accepts color images [three channels (RGB)], and CEUS images are grayscale, so we can input three types of images into one ResNet50 module (referred to here as “pseudo-RGB”). We prepared three ResNet50 modules in parallel so that a total of nine images could be used as input images. Since we prepared 11 types of image datasets (B-mode, six AFs, 1 min, 2 min, 5 min, 10 min), we needed to select nine types from the 11 types. When we created a dataset limited to 2 min after injection, we uniquely used nine datasets (B, six AFs, 1 min, 2 min). When we limited the dataset to 1 min after injection, the B-mode dataset was used twice (B, B, six AFs, 1 min). When creating a dataset limited to 5 or 10 min, the AF dataset was randomly excluded (by Python program), resulting in nine datasets. The input order of the data sets was determined in the order of earliest time phase shown in Fig. 1.

The output of each ResNet50 was fully connected (FC), concatenated into a series of arrays, and combined into a final output of three classes (The number of output channels are also listed on the FC block in Fig. 3). A data augmentation module was used to virtually increase the amount of data. Before augmentation, all nine images (three pseudo-RGB images) were packed into a 4-dimensional image. In this way, the same manner of augmentation works for a set of images. The parameters used for the augmentation were: zoom range [0.8, 1.2], horizontal flip, vertical flip, height shift range [0.1], width shift range [0.1], and rotation range [15 degrees] (keras ImageDataGenerator). This CNN program allowed us to select any nine images from the CEUS dataset. These input images were resized to 244 × 244 pixels. We used a 32 batch size and 600 epochs for training parameters. Cross-validation was performed by 10 separations. We also prepared one pseudo-RGB image input model shown in Fig. 3b for comparison with the standard reference. It was used to evaluate prediction performance when only a single time phase was used. Also, even in the conventional standard method, the ResNet50 model accepts up to three different greyscale images as the model can accept one color (RGB) image. Thus, we regard input of up to three time phases (three grayscale images; Fig. 3b) as the standard model.

### Analysis method (CNN results)

Using the three-class output results, we calculated the percentage of prediction correctness for each combination of phases. We also calculated the sensitivity and specificity between ‘benign’ and ‘malignant’ cases by merging HCC and non-HCC malignancies into ‘malignant’. These accuracy values were also compared with visual scores determined by the observers.

## Results

### Patients and liver nodule characteristics

**Fig. 4:**
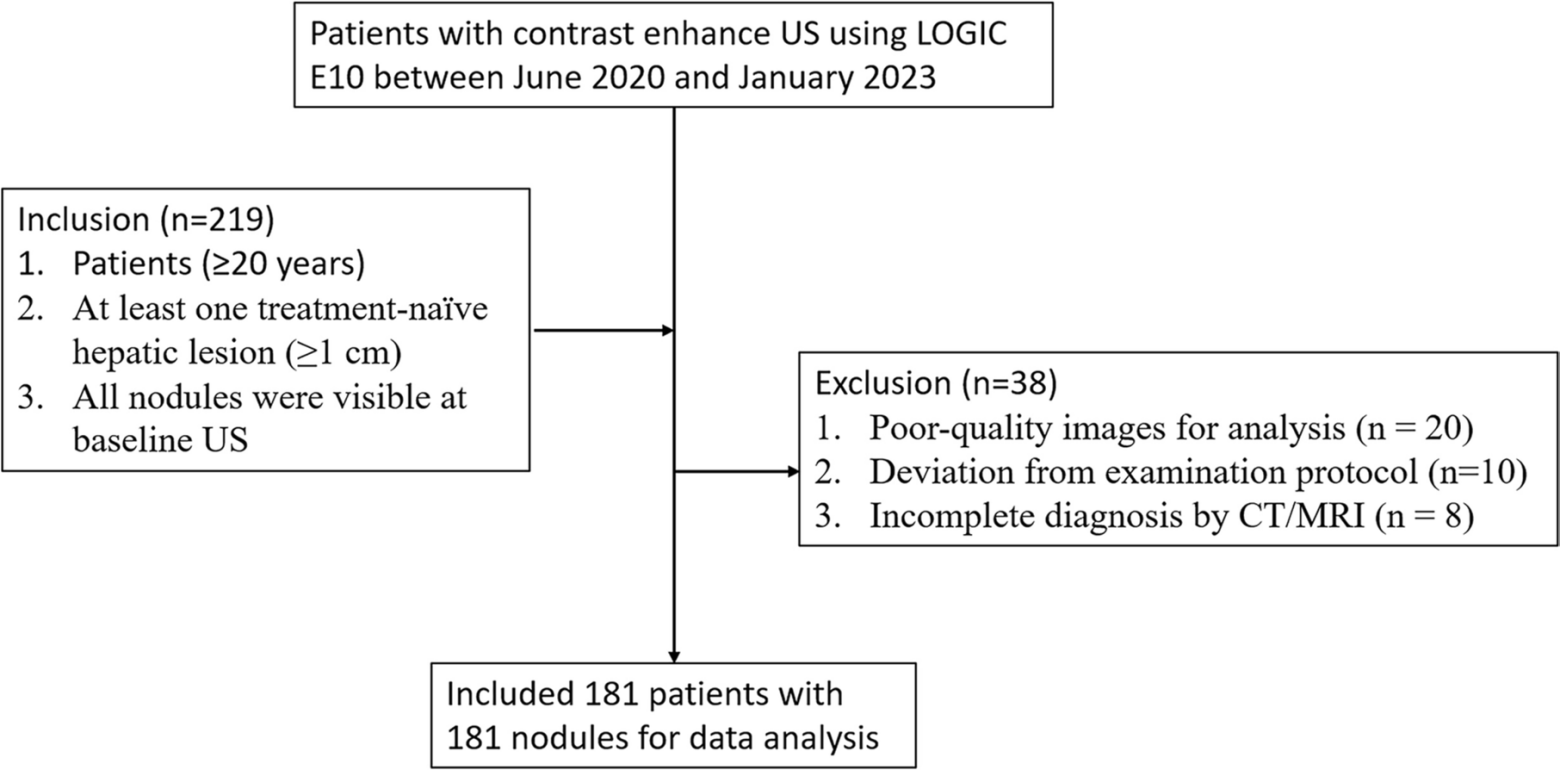
Flow diagram of this study

A total of 219 participants met the inclusion criteria. Among them, 38 were excluded because of poor-quality images for analysis (n = 20), deviation from examination protocol (n = 10), and incomplete diagnosis with CT/MRI (n = 8). Thus, 181 patients with 181 liver nodules (male: 108, female: 73; median age and interquartile range, 68 years and 27–92 years) were ultimately included in this study (Fig. 4). The median size of the observed nodules was 25.0 mm (interquartile range, 17.0–38.5 mm). Of these nodules, 43.1% (78/181) were confirmed as HCCs, 30.4% (55/181) were non-HCC malignancies, and 26.5% (48/181) were benign nodules. Non-HCC malignancies included 38 metastases, 15 intrahepatic cholangiocarcinomas, and two malignant lymphomas. Benign nodules included 25 hemangiomas, 18 focal nodular hyperplasias (FNHs), and five angiomyolipomas (AMLs) (Table 1).

**Table 1:** Clinicopathological characteristics of 181 patients with 181 nodules

Characteristic	Value
Age (years)•	65 ± 15
Sex	
Male	108 (59.7)
Female	73 (40.3)
Median nodule size (mm) •	34 ± 27
Final diagnosis	
HCC	78 (43.1)
Non-HCC malignancy	
Metastases	38 (21.0)
ICC	15 (8.3)
Lymphoma	2 (1.1)
Benign	
Hemangioma	25 (13.8)
Focal nodular hyperplasia	18 (9.9)
Angiomyolipoma	5 (2.8)
Standard reference of diagnosis	
Pathologic diagnosis	138 (76.2)
Noninvasive diagnosis	43 (23.8)

Unless otherwise noted, data are numbers of patients or lesions, with percentages in parentheses

*HCC* hepatocellular carcinoma, *ICC* intrahepatic cholangiocarcinoma

•Data are mean ± standard deviation

### Reference standard of the targeted lesions

Of all the included nodules, all non-HCC malignancies (30.4%: 55 of 181) were diagnosed histopathologically (surgery, n = 5; biopsy, n = 50). Of HCCs, 76.9% (60 of 78) were diagnosed histopathologically (surgery, n = 4; biopsy, n = 56). The remaining 23.1% (18 of 78) of HCCs were noninvasively diagnosed as HCC because they were categorized as LR-5 according to CT/MRI LI-RADS version 2018. All FNHs and AMLs (12.7%: 23 of 181) were diagnosed histopathologically (surgery, n = 0; biopsy, n = 23). Information on hepatic tumor pathology and immunohistochemistry is routinely described in the pathological reports at our institution. All hemangiomas (13.8%: 25 of 181) showed typical findings such as peripheral globular and centripetal enhancement and/or high signal intensity on T2-weighted MRI. Thus, these were regarded as hemangiomas without pathologic confirmation.

### Observer study results

The results of the visual scoring are shown in Table 2. For calculating sensitivity and specificity between “benign” and “malignant” cases, we merged the score of HCC and non-HCC malignant into “malignant”. In general, accuracy increased as the observation time phase increased, regardless of the length of experience of the observer. However, when the image set was limited to 2 min, there was a large difference in accuracy between the expert (R1, R2) and non-expert (R3–R5).

**Table 2:** Visual scoring results for five hepatologists

	B (%)	B-2 m (%)	B-5 m (%)	B-10 m (%)
R1				
Accuracy	66.5	81.3	83.0	90.1
Sensitivity	65.4	85.0	84.2	96.2
Specificity	69.4	71.4	79.6	73.5
R2				
Accuracy	58.2	85.7	90.1	86.3
Sensitivity	48.9	88.7	92.5	84.2
Specificity	83.7	77.6	83.7	91.8
R3				
Accuracy	61.0	65.9	79.7	86.3
Sensitivity	60.2	54.1	77.4	85.7
Specificity	63.3	98.0	85.7	87.8
R4				
Accuracy	68.1	64.8	67.0	81.9
Sensitivity	79.7	57.9	58.6	85.0
Specificity	36.7	83.7	89.8	73.5
R5				
Accuracy	55.5	72.0	79.7	82.4
Sensitivity	45.1	69.2	76.7	82.0
Specificity	83.7	79.6	87.8	83.7

B-1 m: Judged using limited images B-mode only, B-2 m: within 2 min, B-5 m: within 5 min, B-10 m: Judged using entire image set

### Classification results

First, the classification results and diagnostic performance of the conventional one-input ResNet50 model are shown in Table 3 for comparison. The upper left group includes the results obtained using only one dataset in the time phase, while the lower right group includes the results obtained using three datasets. Even in the conventional method, the ResNet50 model accepts three datasets of greyscale images as the model can accept one color (RGB) image. Since the number of combinations is very large, only the top five results in descending order of accuracy are shown.

**Table 3:** Diagnostic performance of conventional one-input CNN

	B (%)	AF (%)	1 m (%)	2 m (%)	5 m (%)	10 m (%)
Benign	20.0	36.7	15.0	40.0	40.0	51.7
HCC	60.4	68.8	66.7	65.6	54.2	61.5
Meta	47.2	25.0	51.4	51.4	54.2	65.3
Average	45.6	46.5	48.2	54.4	50.4	60.1
Accuracy	69.3	70.6	68.9	75.0	73.7	77.6
Sensitivity	86.9	82.7	88.1	87.5	85.7	86.9
Specificity	20.0	36.7	15.0	40.0	40.0	51.7

	B-5-10 (%)	B-1-5 (%)	A-2-10 (%)	1-5-10 (%)	1-2-5 (%)
Benign	53.1	53.1	45.7	50.0	46.9
HCC	65.4	71.8	69.6	75.0	61.5
Meta	61.8	65.5	59.5	75.0	67.3
Average	61.0	64.8	60.2	68.4	59.3
Accuracy	81.9	80.2	79.7	78.9	78.6
Sensitivity	92.5	90.2	92.5	89.3	90.2
Specificity	53.1	53.1	45.7	50.0	46.9

Diagnostic performance of conventional three-input CNN (top eight in descending order of accuracy)

*B* B-mode, *A* arterial flow, *Number* each time phase

Among the single datasets, the average correct answer rate and the accuracy of “10 m” showed the highest value (60.1% and 77. 6% respectively). Regarding the three-input results, the best accuracy score was the combination of “B-5–10 m” (81.9%), which was higher than the highest score of the single dataset. Most of the datasets after the second included 10-min or 5-min datasets, and the scores were higher than any single dataset.

The classification results and diagnostic performance of the proposed nine-input model are shown in Table 4. Each caption in the table means the time range used for input. For example, “B-1 m” means that the input dataset was chosen between B-mode images and 1 min after injection. In other words, “B-1 m” contains the same information as if the examination was completed 1 min after the injection. In this table, we used the datasets under the assumption of a limited examination time. For example, “B-AF” is assumed to be diagnosed with images up to the arterial blood flow phase, i.e., only “B-mode” and “Arterial Flow” datasets were used as input. Similarly, “B-1 m” includes “B-mode”, “Arterial Flow”, and “1 min” datasets (others are the same as above). Thus, “B-10 m” includes the complete image information of the entire time phase up to the Kupffer phase. From the results, “B-10 m” showed the highest average correct answer rate (71.4%) and accuracy (85.7%), which were significantly higher than any results yielded by the conventional model. Similarly, the accuracies of “B-5 m” and “B-2 m” were significantly higher than any results yielded by the conventional model, but “B-1 m” was not superior or inferior to the results of the conventional three-image input model.

**Table 4:** Diagnostic performance of proposed nine-input CNN

	B-AF (%)	B-1 m (%)	B-2 m (%)	B-5 m (%)	B-10 m (%)
Benign	42.9	44.4	61.2	63.3	65.3
HCC	75.6	74.3	73.1	73.1	76.9
Meta	27.3	63.3	61.8	60.0	69.1
Average	52.2	62.8	66.5	66.5	71.4
Accuracy	74.2	79.9	82.4	85.2	85.7
Sensitivity	85.7	93.3	90.2	93.2	93.2
Specificity	42.9	44.4	61.2	63.3	65.3

B: B-mode only, B-AF: input B-mode and arterial flow, B-1 min: input between B-mode and 1 min after injection. B-2 m, B-5 m, B-10 m: same as above

## Discussion

The main purpose of this study was to develop a superior AI model for classifying liver nodules. There have been several deep learning studies that focused on liver nodule classification using CEUS images [14–16, 18, 22]. Since contrast-enhanced diagnostic imaging shows various patterns of blood flow due to the time phase after injection of the contrast agent, some prior reports utilized a multiple-input model, which is a reasonable approach for all imaging modalities such as dynamic CT [23], MR [24, 25], and ultrasound [20]. In the present study, we proposed a new multi-input deep learning model that allows us to accept up to nine phase datasets in parallel. The reason is that the timing of washout is an important feature for tumor classification. Referring to LI-RADS CEUS, typical metastatic tumors are rapidly washed out within 1 min after injection, whereas typical HCCs are slowly and mildly washed out at around 2 min. Also, most benign nodules maintain their enhancement for more than 5 min. This suggests that we need more than three different images, which is not available in the traditional one-input (three grayscale images) model.

Our second aim was to investigate which time phase contributes to identification of each tumor type, which is essential for identification. Sonazoid, a so-called ‘Kupffer cell contrast agent’, accumulates in liver parenchyma. Observation of images 10 min after injection provides more clarity in diagnostic accuracy as malignant nodule defects are more pronounced in the post-vascular phase (aka, Kupffer phase) [26]. However, the examination may take longer. In order to increase examination efficiency, a method to complete diagnosis in a shorter time is desired. We will discuss this point based on our results.

**Fig. 5:**
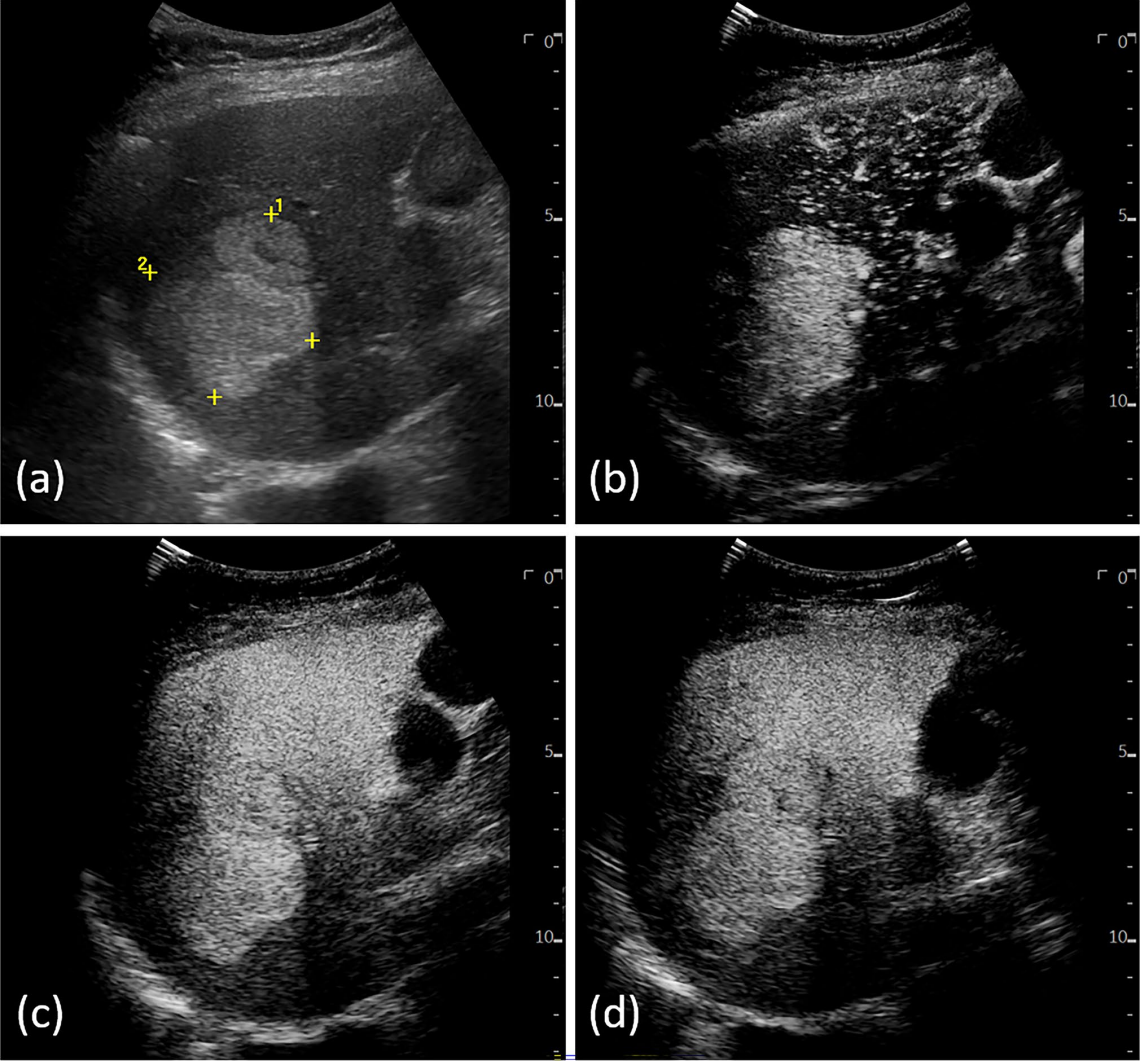
Image example of HCC exhibiting poor washout in the Kupffer phase. **a** B-mode, **b** arterial inflow, **c** 2 min after injection, and **d** 10 min after injection. The nodule looks hyperechoic in B-mode before injection of Sonazoid, and this tissue-harmonic signal affected the image 10 min after injection

Table 3 shows the possibilities and limitations of discrimination with only a single image. Regarding the average correct answer rate, the “10 m” dataset showed the highest rate (60.1%). This is an important finding as we also confirmed that the Kupffer phase imaging pattern intrinsically contributes to liver tumor differentiation even when using a machine learning approach. This is a reasonable feature that the image pattern at the Kupffer phase is considered to be the important time phase. On the other hand, the result of “2 m” was the second highest, even higher than “5 m”. This is also reasonable because “2 m” is the branch point of the three classes, no washout (benign), mild washout (HCC), and marked washout (non-HCC malignant). Of course, there are limits to trying to classify a lesion based on just one phase, but what the AI showed is an important indication that each single phase has different characteristics. From the results of the three inputs (still within a conventional way), there was another finding. As with “10 m”, B-mode images before injection were useful for discrimination. In practice, we sometimes encounter a poor washout image when the tumor is hyper-enhanced before microbubble injection. This brightness is thought to be due to residual tissue harmonic signals that lead to ‘artifact’ brightness even after the contrast agent has been washed out (Fig. 5). The results therefore suggest that the combination of B-mode and enhanced images (mainly “10 m”) may be advantageous in distinguishing these artifacts. A comparison of Tables 3, 4 reveals that our proposed nine-input model has superior performance in terms of accuracy. We can see that the accuracy improves as the types of input datasets increase, which is reasonable as a principle of machine learning. Furthermore, it is worth mentioning that performance of over 82% accuracy was maintained even when using only image datasets of up to 2 min.

Here, we consider the limitations of this study. First, this was a single-center study. As this was the first attempt of our study, it was a complicated protocol involving several redundant steps (e.g., image acquisition every minute from 1 to 10 min). We expect that the findings from this study will be used to simplify the protocol and expand it into a multicenter investigation in the future.

Second, although the accuracy of the proposed method is greater than 85%, higher performance will be required for practical differential diagnosis. In fact, the accuracy of the proposed method using the entire dataset “B-10” was inferior to the visual score of the expert. However, it was known to have the same or better performance than the experts in the case of a dataset limited to 2 min after injection. One of the possible reasons is that the data size was still small considering the typical deep learning approach. Data augmentation is a useful method that can help compensate for the lack of data size and improve performance, so we proposed a unique method to synchronize and extend nine datasets, and we confirmed an improvement in accuracy compared to the conventional model on the same dataset. As we continue to improve our machine learning models, we expect that the learning performance will improve as we increase the amount of CEUS data. Additionally, in this study, AF images were managed as six discrete images. However, the image pattern of contrast agent inflow changes dynamically within seconds. Therefore, in an ideal situation, an AF dataset might need to be managed as a movie clip containing many frames. The potential for increased image information for training should be considered in future studies.

Another reason may lie in the clinical data itself. Table 5 shows the results of visual scoring of the number of washout cases in each time phase. Among visual scoring, the presence or absence of washout is a comparison of the brightness relative to the surrounding tissue, so it can be said that it is a relatively objective judgment rather than a qualitative diagnosis based on visual observation. There are several notable points in this table. (a) In the Kupffer cell phase (10 min after injection), typical malignant nodules should show marked washout, but in the case of combined HCC and non-HCC malignancies, 9.8% had no washout. (b) By referring to LI-RADS CEUS, typical HCCs showed washout at 2 min after injection, but 24.5% of benign nodules showed washout within 1 min. Also, 11 HCCs (14.1%) showed washout within 1 min, which would be scored as LR-M. This fact suggests that the CEUS clinical data we obtained contained more realistically complex patterns. In other words, although we generally use physiological information in addition to images for differential diagnosis, these exceptional patterns may deceive us when judging based on imaging alone. We expect machine learning to learn such exceptions and further improve the score as the data size increases.

**Table 5:** Visual scoring of the number of washouts in each phase

	Number of lesions		Number of	Washout	
		1 min	2 min	5 min	10 min
HCCs	78	11	29	57	66
		(14.1%)	(37.2%)	(73.1%)	(84.6%)
Non-HCC	55	47	52	54	54
Malignancies		(85.5%)	(94.5%)	(98.2%)	(98.2%)
Benign lesions	49	2	12	8	16
		(4.1%)	(24.5%)	(16.3%)	(32.7%)
Total	182				

## Conclusion

In conclusion, we can draw the following two conclusions: (1) The proposed nine-input model is relatively superior to the conventional single-input (three-image) models. (2) Machine learning approaches have the potential to identify tumor types earlier than the Kupffer phase, but at the same time, machine learning confirmed that Kupffer-phase Sonazoid images contain essential information for the classification of liver tumors.

## Data Availability

Analysis data were generated at Tokyo Medical University. Derived data supporting the findings of this study are available from the corresponding author (N.K.) on request.
