# Correction: Deep learning approach for discrimination of liver lesions using nine time-phase images of contrast-enhanced ultrasound

**DOI:** 10.1007/s10396-025-01544-1

**Published:** 2025-04-15

**Authors:** Naohisa Kamiyama, Katsutoshi Sugimoto, Ryuichi Nakahara, Tatsuya Kakegawa, Takao Itoi

**Affiliations:** 1 Ultrasound General Imaging, GE HealthCare Japan, 127 Asahigaoka-4, Hino, Tokyo 191-0065 Japan; 2 Department of Gastroenterology and Hepatology, Tokyo Medical University, Tokyo, 160-0023 Japan; 3 Department of Orthopedic Surgery, Dentistry and Pharmaceutical Sciences, Okayama University Graduate School of Medicine, Okayama, 700-8558 Japan

**Correction: Journal of Medical Ultrasonics (2023) 51:83–93** 10.1007/s10396-023-01390-z

The article “Deep learning approach for discrimination of liver lesions using nine time‑phase images of contrast‑enhanced ultrasound”, written by Naohisa Kamiyama, Katsutoshi Sugimoto, Ryuichi Nakahara, Tatsuya Kakegawa and Takao Itoi, was originally published under exclusive license to The Author(s), under exclusive licence to The Japan Society of Ultrasonics in Medicine. As a result of the subsequent decision to publish the article under the open access model, the article’s copyright notice was changed on 1 April 2025 to © The Author(s) 2023 and the article is now distributed under a Creative Commons Attribution 4.0 International License, which permits use, sharing, adaptation, distribution and reproduction in any medium or format, as long as you give appropriate credit to the original author(s) and the source, provide a link to the Creative Commons licence, and indicate if changes were made. The images or other third party material in this article are included in the article’s Creative Commons licence, unless indicated otherwise in a credit line to the material. If material is not included in the article’s Creative Commons licence and your intended use is not permitted by statutory regulation or exceeds the permitted use, you will need to obtain permission directly from the copyright holder. To view a copy of this licence, visit http://creativecommons.org/licenses/by/4.0

The original article has been corrected.

